# Risk of premature cardiovascular disease and all-cause mortality in young adults, association with risk factor prevalence early in life

**DOI:** 10.1186/s12872-025-04814-5

**Published:** 2025-05-07

**Authors:** Chenrui Zhu, Liuxin Li, Mingchen Zhao, Jie Li, Haibo Gao, Huiying Li, Yan Liu, Chunpeng Ji, Zhe Huang

**Affiliations:** 1 Department of Cardiology, Kailuan General Hospital, 57 Xinhua East Road, Tangshan, 063000 China; 2 Department of Clinical Epidemiology, Shengjing Hospital of China Medical University, Shenyang, China; 3 Liaoning Key Laboratory of Precision Medical Research On Major Chronic Disease, Shenyang, China; 4 Department of General Practice, Kailuan General Hospital, 57 Xinhua East Road, Tangshan, 063000 China; 5 Department of Cardiology, The Affiliated Hospital of North China University of Science and Technology, Tangshan, 063000 China

**Keywords:** Cardiovascular disease, All-cause mortality, Young adult, Joint risk factor, Cohort study

## Abstract

**Background:**

With the increase in risk factors and the emergence of unhealthy lifestyles in young adults, we need to pay more attention to the cardiovascular health of this group. This study aimed to assess the association of the degree of joint risk factor control with premature cardiovascular disease (CVD) and all-cause mortality in young people.

**Methods:**

*Kailuan Study* is a prospective cohort study based on a community population, which began in June 2006, and followed up every two years. A sample of 16,519 eligible participants in the *Kailuan* cohort was recruited in this current study and 15,948 was included in the final analysis, with an average age of 32.34 ± 5.19 years, and a male proportion of 74.76%. Based on the control status of the risk factors, participants were divided into three groups: well-controlled group (≥ 7 risk factors controlled), moderately controlled group (5–6 risk factors controlled), and poorly controlled group (≤ 4 risk factors controlled). Multivariate Cox proportional hazard model was used to analyse the relationship between the joint control of risk factors and onset of CVD and all-cause mortality.

**Results:**

During a mean follow-up period of 14.78 ± 1.33 years, we identified 285 incident CVD cases and a total of 274 deaths from all causes. Compared to the well-controlled group, the moderately controlled group and poorly controlled group exhibited progressively higher risks of CVD and all-cause mortality. The adjusted hazard ratios (HRs) for CVD in the moderately controlled group and poorly controlled group were 2.24 (95% confidence interval [CI]: 1.66–3.02) and 3.09 (95% CI: 2.04–4.68), respectively. The adjusted HRs for all-cause mortality in these two groups were 1.53 (1.15–2.04) and 2.65 (1.79–3.92), respectively.

**Conclusions:**

We observed an inverse relationship between the degree of risk factor control and the risk of CVD and all-cause mortality in young adults, emphasizing the importance of actively controlling more risk factors in early life.

**Supplementary Information:**

The online version contains supplementary material available at 10.1186/s12872-025-04814-5.

## Introduction

A large number of studies have indicated that a favourable cardiovascular health (CVH) status in middle-aged and elderly population is significantly associated with a substantial reduction in cardiovascular disease (CVD) and mortality rates [1–4]. However, there has been limited research focusing on the CVH status of young people. Some studies suggested that CVH measured during adolescence or early adulthood is correlated with later subclinical disease markers, including increased carotid intima-media thickness, left ventricular hypertrophy and diastolic dysfunction [5, 6]. Although intimal-medial thickness [7], left ventricular hypertrophy [8, 9] and diastolic dysfunction [10, 11] are all related to cardiovascular events and mortality, there is still a lack of direct research on the relationship between CVH status and clinical outcomes in young people. This often leads to the neglect of cardiovascular health issues in this population. Early emergence of risk factors such as elevated blood pressure, blood glucose, lipid levels, increased body mass index (BMI), and unhealthy lifestyles within this group may significantly increase the risk of early-onset cardiovascular diseases in the future.

In order to alleviate the burden of CVD, the American Heart Association (AHA) formulated and updated the concept of CVH in 2022. They introduced the"Life's Simple 8"(LE8), which involves the simultaneous presence of four health behaviors (diet, physical activity, smoking, and sleep) and four health factors (BMI, blood lipid, blood glucose and blood pressure) [12, 13]. Studies have indicated that effective control of these factors can significantly reduce the incidence and mortality of CVD [1–3]. However, previous research has predominantly focused on the middle-aged and elderly population. Our study, on the other hand, examined the associations between the number of risk factors under control based on the LE8 in aged ≤ 40 years young adults and the occurrence of premature CVD and all-cause mortality.

## Methods

### Study design and study population

**Fig. 1:**
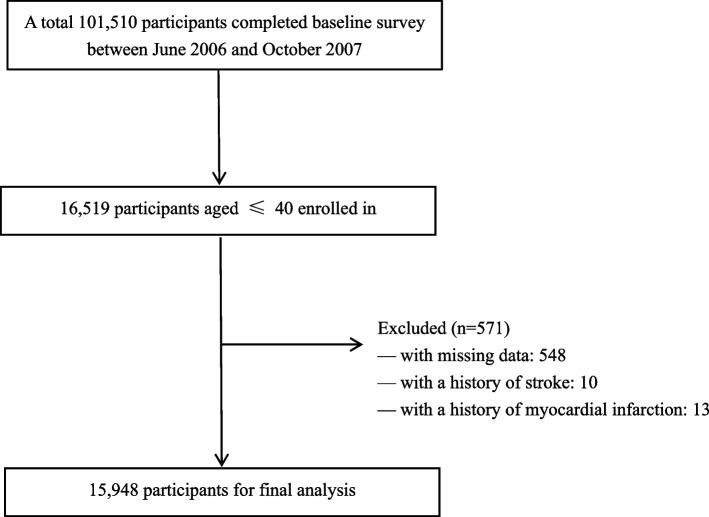
Flow diagram of the participants selection

*Kailuan Study* [14, 15] (Registration No.: ChiCTR-TNRC-11001489) is a prospective cohort study based on a community population in Tangshan, a city in northern China. From 2006 to 2007, all employees and retirees of Kailuan Group received the first physical examination in Kailuan General Hospital and its 11 affiliated hospitals. A total of 101,510 participants completed baseline survey between June 2006 and October 2007, 16,519 participants aged ≤ 40 years were enrolled. After excluding participants with missing data (*n* = 548) and those with a history of stroke (*n* = 10) or myocardial infarction (*n* = 13), 15,948 individuals were included in the final analysis (Fig. 1).

The study was performed according to the guidelines of the Helsinki Declaration and was approved by the ethics committee of *Kailuan* General Hospital (2006–05). All participants provided written informed consent.

### Data collection

The data included questionnaire assessments, physical examinations, and laboratory tests, which were updated every two years. Standardized questionnaire data, including socio-demographic factors, health and medication status, and lifestyle information, were collected by trained professionals via face-to-face interviews. Height, weight, and blood pressure were measured by trained nurses. BMI was calculated as weight in kilograms divided by height in metres squared.

Fasting venous blood samples (5 ml) were collected from the cubital vein between 7:00 and 9:00 on the day of the examination. Biochemical indicators, including fasting blood glucose (FBG), total cholesterol (TC), and high-density lipoprotein cholesterol (HDL-C), were analysed by an automatic analyser (Hitachi 747; Hitachi, Japan) at the *Kailuan* Hospital Central Laboratory. Non-high-density lipoprotein cholesterol (non-HDL-C) was calculated by subtracting HDL-C from TC.

### Joint risk factor assessment and classification

In this study, we assessed eight important modifiable risk factors and developed the risk factor scoring criteria based on the AHA “LE8” standards (Table S1). The LE8 score includes four lifestyle factors (diet quality, physical activity, smoking status, and sleep quality), and four health factors (BMI, non-high density lipoprotein cholesterol [non-HDL cholesterol], blood glucose, and blood pressure). Due to the lack of detailed dietary data, we used the intake of salt, tea, and high-fat foods as surrogate indicators for dietary quality. Previous reports have confirmed the association of these indicators with the occurrence of CVD in the Chinese population [16–18]. Each of the eight risk factors is scored on a scale of 0 to 100.

Degree of risk factor control: each risk factor scoring ≥ 50 points was considered controlled, while risk factor scoring < 50 points was considered not controlled. In this current study, based on the control status of the eight risk factors, participants were divided into three groups: well-controlled group (≥ 7 risk factors controlled), moderately controlled group (5–6 risk factors controlled), and poorly controlled group (≤ 4 risk factors controlled).

### Assessment of outcomes

The primary outcomes of the study were incident CVD and all-cause mortality. The types of CVD included stroke (ischemic stroke and hemorrhagic stroke, ICD-10: I61-I64) and myocardial infarction (ICD-10: I21-I22).The outcomes in the prospective cohort were updated via medical insurance information and searching for hospital records until December 31, 2021. Stroke was diagnosed according to the World Health Organization's criteria, based on neurological signs, clinical symptoms, and neuroimaging examinations, including computerized tomography scans or magnetic resonance imaging [19]. Myocardial infarction was diagnosed referring to the 4 th universal definition of myocardial infarction (2018) [20]. Mortality was collected from provincial vital statistics offices. All participants were followed up every two years to collect data on CVDs and mortality. The follow-up for deaths was not terminated by CVDs.

### Statistical analyses

Continuous variables were described as means ± SD and were compared using ANOVAs. Categorical variables were described as percentages and were compared using χ^2^ tests. Kaplan–Meier curves were drawn and cumulative incidence rates of CVD, all-cause mortality, stroke and myocardial infarction in different groups were observed. Multivariate Cox proportional hazard regression models were performed to analyse the association of different control levels of risk factors and each additional uncontrolled risk factor with CVD and all-cause mortality. The model corrected for age, gender, education, income, marital status, alcohol consumption and family history of CVD. The proportional hazards assumption for the Cox model was tested using the Schoenfeld residuals method, and no violations were observed. To test the robustness of the results, sensitivity analysis was undertaken. First, we excluded participants who experienced CVD or death within the two years of follow-up, and those who had a history of cancer at baseline or onset cancer during follow-up. Second, we postponed the occurrence of CVD or death by 2 years for further analysis to avoid the randomness of events.

All analyses were conducted using SAS version 9.3 (SAS Institute, Inc., Cary, NC). A two-sided *p* value < 0.05 was considered statistically significant in the current study.

## Results

### Baseline participant characteristics

There were 15,948 individuals included in the final analysis, with 7,708 participants in the well-controlled group, 7,239 participants in the moderately controlled group, and 1,001 participants in the poorly controlled group. The average age was 32.34 ± 5.19 years, with a male proportion of 74.76% and 47.16% having a senior high school education or above. In groups with fewer controlled risk factors, participants tended to be older and have lower incomes. Additionally, we observed greater proportions of males, individuals with a senior high school education or above, individuals who were married, and individuals with a family history of CVD in these groups, while the percentage of nondrinkers was lower in the poorly controlled group than in the other groups (Table 1).

**Table 1:** Baseline characteristics of the study population

**Characteristics**	**Total** (*N* = 15,948)	**Degree of Risk Factor Control**			***P* value**
		** ≥ 7 Risk Factors** (*N* = 7708)	**5–6 Risk Factors** (*N* = 7239)	** ≤ 4 Risk Factors** (*N* = 1001)	
**Age (y)**	32.34 ± 5.19	31.87 ± 5.19	32.64 ± 5.19	33.82 ± 5.19	< 0.001
**Male (%)**	11,923(74.76)	4541(58.91)	6406(88.49)	976(97.50)	< 0.001
**Education level (%)**					
Illiteracy or primary school	148(0.93)	34(0.44)	78(1.08)	36(3.60)	< 0.001
Junior high school	8279(51.91)	3567(46.28)	4109(56.76)	603(60.24)	< 0.001
Senior high school and above	7521(47.16)	4107(53.28)	3052(42.16)	721(72.03)	< 0.001
**Income level, ¥/month (%)**					
≤ 800	12,827(80.43)	6040(78.36)	5952(82.22)	835(83.42)	< 0.001
800–1000	1574(9.87)	753(9.77)	718(9.92)	103(10.29)	< 0.001
≥ 1000	1547(9.70)	915(11.87)	569(7.86)	63(6.29)	< 0.001
**Alcohol consumption (%)**					
Never	8168(51.22)	4807(62.36)	3106(42.91)	255(25.47)	< 0.001
Current drinker	7496(47.00)	2788(36.17)	3987(55.08)	25(2.50)	< 0.001
Past	284(1.78)	113(1.46)	146(2.01)	721(72.03)	< 0.001
**Married individuals (%)**	14,477(90.78)	6856(88.95)	6668(92.11)	953(95.20)	< 0.001
**Family history of CVD (%)**	816(5.12)	337(4.37)	388(5.36)	91(9.09)	< 0.001
**Health factors under controlled (%)**					
Blood glucose	15,540(97.44)	7697(99.86)	7003(96.74)	840(83.92)	< 0.001
Blood pressure	12,800(80.26)	7589(98.46)	4906(67.77)	305(30.47)	< 0.001
Blood lipids	13,459(84.39)	7522(97.59)	5546(76.61)	391(39.06)	< 0.001
Body mass index	14,557(91.28)	7632(99.01)	6348(87.69)	577(57.64)	< 0.001
**Health behaviours under controlled (%)**					
Diet health	4582(28.73)	3063(39.74)	1372(18.95)	147(14.69)	< 0.001
Nicotine exposure	10,051(63.02)	6604(85.68)	3251(44.91)	196(19.58)	< 0.001
Physical activity	14,134(88.63)	7566(98.16)	6035(83.37)	533(53.25)	< 0.001
Sleep health	15,134(94.90)	7635(99.05)	6740(93.11)	759(75.82)	< 0.001

### Associations between the degree of joint risk factor control and CVD and all-cause mortality

**Fig. 2:**
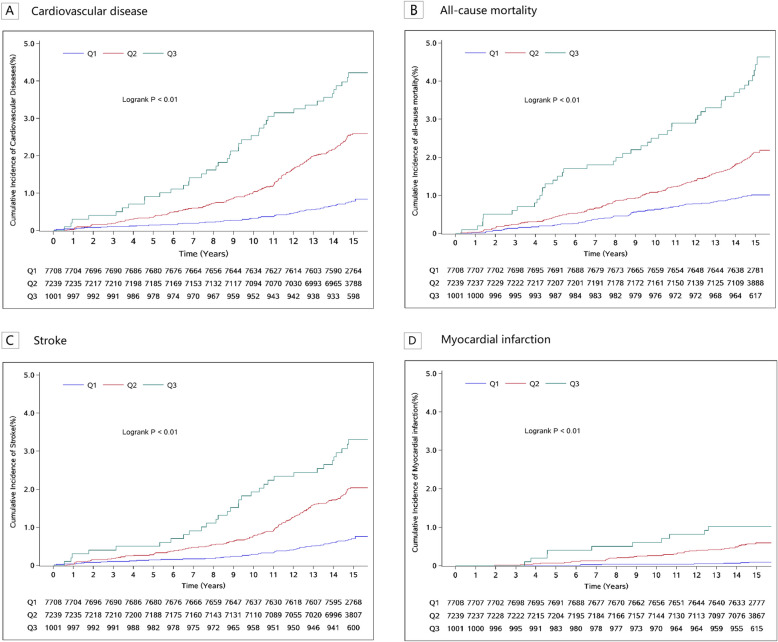
Kaplan–Meier curves for the incidence of CVD, all-cause mortality, stroke, and myocardial infarction and the degree of joint risk factor control. Caption: Q1: ≥ 7 risk factors controlled, Q2: 5–6 risk factors controlled, Q3: ≤ 4 risk factors controlled. Cox model was adjusted for age, sex, education level, income level, marital status, alcohol consumption and family history of CVD. CVD, cardiovascular diseases

Dring a mean follow-up period of 14.78 ± 1.33 years, 285 new CVD cases (231 cases of stroke and 59 cases of myocardial infarction) and a total of 274 deaths from all causes were observed. The incidence rates of CVD and all-cause mortality in the well-controlled group, moderately controlled group and poorly controlled group were 0.53 and 0.67, 1.72 and 1.42, 2.82 and 2.97 per 1000 person-years, respectively. (Fig. 2, log-rank test, p < 0.01). We observed significant inverse associations between the degree of risk factor control and the risk of incident CVD and all-cause mortality (Table 2). The risks of CVD in the moderately controlled group and poorly controlled group were 2.24 times (95% CI: 1.66–3.02) and 3.09 times (95% CI: 2.04–4.68) higher respectively compared to that of the well-controlled group. The risks of all-cause mortality in the moderately controlled group and poorly controlled group were also significantly increased, with HR values of 1.53 (95% CI:1.15–2.04) and 2.65 (95% CI:1.79–3.92), respectively. For each additional uncontrolled risk factor, the risks of CVD and all-cause mortality increased by 1.44 times (95% CI: 1.30–1.60) and 1.30 times (95% CI: 1.17–1.45), respectively (Table 2).

**Table 2:** Association between the degree of joint risk factor control and incident CVD and all-cause mortality

	**Degree of Risk Factors Control**			***P* for trend**	**Each Additional Uncontrolled Risk Factor**
	** ≥ 7 Risk Factors** (*N* = 7708)	**5–6 Risk Factors** (*N* = 7239)	** ≤ 4 Risk Factors** (*N* = 1001)		
**CVD**					
Events/person-years	60/114140	184/106972	41/14556		
Incidence rate	0.53	1.72	2.82		
Model 1	1 (reference)	2.29(0.70–3.09)	3.15(2.07–4.73)	< 0.001	
Model 2	1 (reference)	2.24(1.66–3.02)	3.09(2.04–4.68)	< 0.001	1.44(1.30–1.60)
**All-cause mortality**					
Events/person-years	77/114446	153/107832	44/14800		
Incidence rate	0.67	1.42	2.97		
Model 1	1 (reference)	1.58(1.19–2.10)	2.78(1.89–4.08)	< 0.001	
Model 2	1 (reference)	1.53(1.15–2.04)	2.65(1.79–3.92)	< 0.001	1.30(1.17–1.45)

Incidence rate: per 1000 person-years

Model 1: adjusted for age, sex

Model 2: Model 1 + education level, income level, marital status, alcohol consumption and family history of CVD

### Subgroup analyses

We categorized CVD into stroke and myocardial infarction, and observed the association of varying degrees of risk factor control. As presented in Fig. 1, the incidence rates of stroke and myocardial infarction showed statistical differences among the three groups, with the lowest incidence rate observed in the well-controlled group. The risks of incident stroke and myocardial infarction in the moderately controlled and poorly controlled groups were both higher compared to those of the well-controlled group. Stroke was divided into ischemic stroke and hemorrhagic stroke, and the results were consistent with the above. For each additional risk factor uncontrolled, the risks of stroke and myocardial infarction increased by 1.39 times (95% CI: 1.24–1.56) and 1.67 times (95% CI: 1.34–2.07), respectively (Table S2).

To clarify whether the effect of degrees of risk factors control on CVD and all-cause mortality is age- and sex-dependent, Cox regression models were conducted after stratification by age and sex. As presented in Table S5, the interaction between age, sex, and control levels of risk factors was not statistically significant (*P* for interaction = 0.67 and 0.34, respectively). This suggests that the association between control levels of risk factors and cardiovascular disease (CVD) is independent of age and sex, implying that the impact of risk factor control on CVD outcomes does not vary significantly across different age groups or between males and females.

### Distinct effects of health factors and health behaviors on CVD and all-cause mortality

After categorizing all risk factors into health factors and health behaviors, our study found that health factors had a greater impact on outcomes compared to health behaviors (Table S3). For each reduction of a controlled health factor, the risk of CVD and all-cause mortality increased by 1.75 times (95% CI:1.55–1.98) and 1.38 times (95% CI:1.21–1.58), respectively, and the risks of stroke and myocardial infarction increased by 1.69 times (95% CI:1.47–1.93) and 2.10 times (95% CI:1.63–2.69), respectively. For each reduction of a controlled health behavior, only the risk of all-cause mortality increased by 18%.

### Sensitivity analyses

In the sensitivity analyses, HRs for CVD and all-cause mortality associated with the different groups remained robust after excluding participants with CVD events or deaths within the first 2 years of follow-up, and participants with a history of cancer at baseline or cancer onset during follow-up. We also conducted a 2-year lag analysis for associations of duration of adherence, and the results remained consistent with the primary analysis (Table S4). In the existing population, we redefined the degree of risk factor control for sensitivity analysis. Each risk factor scoring ≥ 80 points was considered controlled, while risk factor scoring < 80 points was considered not controlled. We observed no change in the conclusion that the worse the control of risk factors, the higher the risk of incident cardiovascular disease and all-cause mortality (Table S8). Then we utilized a logistic regression model to calculate the overall estimated propensity scores for eight indicators. Based on these propensity scores, we further computed the inverse probability weighting (IPW) and applied this weighting to the weighted Cox regression analysis to balance the influence of each risk factor, and the results remained consistent with the primary analysis (Table S9).

## Discussion

In this prospective cohort study, we found an inverse relationship between the degree of risk factor control and the risk of premature CVD and all-cause mortality in young adults. The greater the number of controlled factors, the lower the risk of early-onset CVD and all-cause mortality. Our results underscore the importance of better controlling risk factors in the early stages of life to prevent premature cardiovascular events and all-cause mortality.

During 14.8-year follow-up, this current study revealed that in the young population, participants with ≤ 4 controlled risk factors face a 2.09-fold higher risk of cardiovascular events and a 1.65-fold higher risk of all-cause mortality compared to those with ≥ 7 controlled risk factors. Additionally, among participants with 5–6 controlled risk factors, the risks of cardiovascular events and all-cause mortality still increased by 1.24 times and 53%, respectively, compared to those in the well-controlled group. In the subgroup analysis, a close association was observed between the degree of risk factor control and the incidence of stroke and myocardial infarction in the young population. With each additional uncontrolled factor, the risk of cardiovascular events increased by 44%, and the risk of all-cause mortality increased by 30%. Other studies have found that there is a negative correlation between CVH scores and the intima-media thickness of the carotid artery [5, 21], and higher baseline CVH scores are associated with lower risks of early-onset CVD and mortality [22, 23], in young adults. This current study updated the CVH standards from AHA and classified based on the number of risk factors under control, providing new evidence for exploring the relationship between CVH in young populations and early-onset CVD.

We also found that, in the young population, with each exclusion of a controlled health factor (blood pressure, non-HDL-C, FBG, and BMI), there is a significant increase in the risk of CVD, all-cause mortality, stroke and myocardial infarction. A prospective cohort study in the United States, encompassing six large communities, indicated that elevated blood pressure and low-density lipoprotein cholesterol in young adults are associated with an increased risk of late-life cardiovascular diseases [24]. Coronary Artery Risk Development in Young Adults (CARDIA) study also found that higher baseline triglyceride-glucose (TyG) index levels and higher long-term trajectory of TyG index during young adulthood are significantly associated with an increased risk of incident CVD events and all-cause mortality in later life [25]. A UK-based case–control study also indicated that, in a population with an average age of 33, the all-cause mortality rate is significantly higher in individuals with diabetes compared to those without [26]. Another study suggested that weight gain in aged 18–35 group is associated with coronary artery calcification in midlife, indicating a causal association between weight gain in young individuals and subsequent cardiovascular diseases [27]. We also observed that with each exclusion of a controlled health behavior, there is an increased risk of all-cause mortality, without a corresponding increase in the risk of CVD. Possible reasons may be that health behavior were more likely to be impacted by modern work and lifestyle, such as long work time and extensive use of social media, and the effect of behaviour on CVD may be modified by risk factors.

This current study has significant clinical implications from the perspectives of predictive, preventive and precision medicine. Early control of CVD-risk factors in young adults can effectively reduce the risk of premature CVD and all-cause mortality, and greatly alleviate the burden on individuals and the society on the occasion of an aging society. Therefore, it is crucial to emphasize the monitoring and treatment of existing or newly emerging risk factors in the young population, strengthen health education tailored for young individuals, and promote lifestyle improvements. Furthermore, the degree of control of risk factors in the young population may serve as an effective intermediate or surrogate indicator for determining the factors contributing to premature CVD. Given the observed associations between diet, lifestyle, and health outcomes in the cohort, there may be opportunities for public health interventions targeted at improving dietary patterns and promoting healthier lifestyles in young population. For example, policies aimed at increasing access to healthy foods, promoting physical activity, and reducing occupational stress could contribute to improving overall health outcomes. Future research should investigate the mechanisms through which dietary patterns influence health, such as through gut microbiota, inflammation, or metabolic pathways, to better inform targeted interventions. Additionally, investigating how different social determinants of health, such as income, education, and healthcare access, interact with dietary behaviors to impact health outcomes will provide a more comprehensive understanding of the factors that contribute to health disparities.

The limitations of this study need to be addressed. Firstly, the cohort in this study is composed of employees from the Kailuan Group, which may limit the generalizability of the findings to the broader young adult population. This limitation arises from the specific demographic and occupational characteristics of the Kailuan Group employees, which may not reflect the diversity in lifestyle, socio-economic status, or health behaviors found in the general population. The validation in another cohort from different settings would benefit the generalization of the outcomes of the current study. Secondly, there was a lack of representativeness of females in this cohort. This Kailuan cohort study will provide further evidence in the follow-up investigation. Third, for dietary indices, due to the lack of corresponding items in the questionnaire, we opted for salt, tea, and high-fat diet closely associated with Chinese cardiovascular health as substitute indicators for the DASH-style diet [4, 18, 28, 29]. The accuracy of dietary contributions to CVD risk is thus potentially compromised by the use of these limited indicators. However, the findings of this study can be served as a starting point for further research using more detailed and comprehensive dietary data. Lastly, due to the extended follow-up period, potential for participant attrition exists. However, the Kailuan study will deploy an expert panel to review annual discharge records from 11 local hospitals to identify suspected cases of CVD among Kailuan study participants. This measure aims to mitigate the potential impact of participant relocation during the study period on the research outcomes.

## Conclusions

The current study found a clear inverse relationship between the degree of control of risk factors and the risk of CVD as well as all-cause mortality in young adults. Individuals with fewer controlled risk factors are associated with a higher risk of CVD and all-cause mortality. The findings suggested that implementing aggressive primordial preventive strategies to control more risk factors may help to prevent such adverse events in young adults.

## Supplementary Information

Supplementary Material 1.

## Data Availability

No datasets were generated or analysed during the current study.

## Abbreviations

CVD: Cardiovascular disease
HR: Hazard ratio
CI: Confidence interval
CVH: Cardiovascular health
BMI: Body mass index
AHA: American Heart Association
LE8: Life's Simple 8
FBG: Fasting blood glucose
TC: Total cholesterol
HDL-C: High-density lipoprotein cholesterol
non-HDL-C: Non-high-density lipoprotein cholesterol
