# Supplementary material for: Risk of premature cardiovascular disease and all-cause mortality in young adults, association with risk factor prevalence early in life
Source: BMC Cardiovasc Disord. 2025 May 7;25:352. doi: 10.1186/s12872-025-04814-5 (PMC12057125; doi:10.1186/s12872-025-04814-5)
Supplement: Supplementary file 1 — Supplementary Material 1. [file 12872_2025_4814_MOESM1_ESM.docx]

**Supplemental Tables**

[Table S1. Definition and scoring approach for risk factors based on the AHA “LE8” standards 2](#_Toc2417)

[Table S2. Associations between the degree of joint risk factor control and the incidence of stroke and myocardial infarction 5](#_Toc12143)

[Table S3. Distinct effects of health factors and health behaviors on CVD and all-cause mortality (for each additional uncontrolled risk factor) 6](#_Toc16247)

[Table S4. Sensitivity analyses of associations between the degree of joint risk factor control and CVD and all-cause mortality 7](#_Toc11142)

[Table S5. Associations between the degree of joint risk factor control and CVD and all-cause mortality stratified by age or sex](#_Toc11142)..............................................................8

Table S6. Baseline characteristics of the study population (No-missing data and missing data population) .........................................................................................9

Table S7. Association between the degree of joint risk factor control and incident CVD and all-cause mortality (including the population with missing data) ..........10

Table S8. Association between the degree of joint risk factor control and incident CVD and all-cause mortality ( sensitivity analysis of redefining grouping threshold) ..............................................................................................................11

Table S9. Association between the degree of joint risk factor control and incident CVD and all-cause mortality: Results from weighted Cox regression analysis..................................................................................................................12

| **Table S1.** Definition and scoring approach for risk factors based on the AHA “LE8” standards | | | |
| --- | --- | --- | --- |
| Domain | Metric | Method of measurement | Scoring criteria |
| Health behaviours | Diet  health | Measurement: Self-reported intake of salt, fatty foods, and tea.  Example of salt intake measurement: “Which flavor do you prefer?”  Example of fatty foods intake measurement: “How often do you eat fatty foods?”  Example of tea intake measurement: “How often do you drink tea?” | Metric: The unweighted average of salt, fatty foods, and tea scoring.  Salt scoring:  Points Level  100 < 6 g/day  50 6-12 g/day  0 > 12 g/day  Fatty foods scoring:  Points Level  100 < 1 time/week  50 1-3 times/week  0 >3 times/week  Tea scoring:  Points Level  100 ≥ 4 times/week  75 1-3 times/week  50 1-3 times/month  25 < 1 time/month  0 Never |
|  | Physical activity | Measurement: Self-reported time of physical activity per week.  Example for measurement: “How much time do you usually spend on physical activity per week?” | Metric: Minutes of physical activity per week.  Scoring:  Points Level  100 ≥ 80 min  50 20-60 min  0 < 20 min |
|  | Nicotine exposure | Measurement: Self-reported use of cigarettes.  Example for measurement: “Are you currently smoking?” “How many cigarettes do you smoke per day?” | Metric: Smoking status  Scoring:  Points Status  100 Never smoke  50 Former smoker, quit ≥ 1 y  25 Current smoker, < 1 cigarette/d  0 Current smoker, ≥ 1 cigarette/d |
|  | Sleep health | Measurement: Self-reported average hours of sleep per night  Example for measurement: “On average, how many hours of sleep do you get per night?” | Metric: Average hours of sleep per night  Scoring:  Points Level  100 7- < 9 h  90 9- < 10 h  70 6- < 7 h  40 5- < 6 h or ≥ 10 h  20 4- < 5 h  0 < 4 h |
| Health  factors | Body mass index | Measurement: Body weight (kg) divided by height squared (m²).  Example tools for measurement: Objective measurement of height and weight | Metric: Body mass index (kg/m^2^)  Scoring:  Points Level  100 <23  75 23.0-24.9  50 25.0-29.9  25 30.0-34.9  0 >35 |
|  | Blood lipids | Measurement: Plasma total cholesterol minus high-density lipoprotein (HDL) cholesterol.  Example tools for measurement: Fasting blood sample | Metric: Non-HDL cholesterol (mmol/L)  Scoring:  Points Level  100 < 3.36  60 3.36-4. 13  40 4.14-4.90  20 4.91-5.68  0 ≥ 5.69  Subtract 20 points if the treated level. |
|  | Blood glucose | Measurement: Fasting blood glucose (FBG).  Example tools for measurement: Fasting blood sample | Metric: FBG (mmol/L)  Scoring:  Points Level  100 No diabetes with FBG < 5.6  60 No diabetes with FBG 5.6-6.9  40 Diabetes with FBG < 8.6  30 Diabetes with FBG 8.6-10. 1  20 Diabetes with FBG 10.2-11.6  10 Diabetes with FBG 11.7-13.2  0 Diabetes with FBG ≥ 13.3 |
|  | Blood pressure | Measurement: Appropriately measured systolic and diastolic blood pressure.  Example tools for measurement: Corrected mercury sphygmomanometer | Metric: Systolic and diastolic blood pressure (mmHg)  Scoring:  Points Level  100 < 120 / < 80  75 120-129 / < 80  50 130-139 and/or 80-89  25 140-159 and/or 90-99  0 ≥ 160 and/or ≥ 100  Subtract 20 points if the treated level. |

| **Table S2.** Associations between the degree of joint risk factor control and the incidence of stroke and myocardial infarction | | | | | |
| --- | --- | --- | --- | --- | --- |
|  | **Degree of Risk Factor Control** | | | ***P* value** | **Each Additional Uncontrolled Risk Factor** |
|  | **≥7 Risk Factors**  (n=7708) | **5-6 Risk Factors**  (n=7239) | **≤4 Risk Factors**  (n=1001) |  |  |
| **Stroke** | | | | | |
| Events/Person-years | 54/114171 | 145/107154 | 32/14621 |  |  |
| Incidence rate | 0.47 | 1.35 | 2.19 |  |  |
| Model 1 | 1 (reference) | 2.02(1.46-2.78) | 2.72(1.74-4.27) | ＜0.001 |  |
| Model 2 | 1 (reference) | 1.96(1.42-2.71) | 2.66(1.68-4.20) | ＜0.001 | 1.39(1.24-1.56) |
| **Ischemic stroke** | | | | | |
| Events/Person-years | 46/114243 | 118/107350 | 27/14649 |  |  |
| Incidence rate | 0.40 | 1.10 | 1.84 |  |  |
| Model 1 | 1 (reference) | 1.95(1.37,2.77) | 2.74(1.68,4.46) | ＜0.001 |  |
| Model 2 | 1 (reference) | 1.90(1.33,2.70) | 2.66(1.62,4.38) | ＜0.001 | 1.43(1.26,1.63) |
| **Hemorrhagic stroke** | | | | | |
| Events/Person-years | 9/114373 | 32/107614 | 6/14772 |  |  |
| Incidence rate | 0.08 | 0.30 | 0.41 |  |  |
| Model 1 | 1 (reference) | 2.42(1.14,5.10) | 2.70(0.95,7.68) | ＜0.001 |  |
| Model 2 | 1 (reference) | 2.37(1.12,5.03) | 2.65(0.92,7.69) | ＜0.001 | 1.24(0.95,1.61) |
| **Myocardial infarction** | | | | | |
| Events/Person-years | 7/114414 | 42/107641 | 10/14732 |  |  |
| Incidence rate | 0.06 | 0.39 | 0.68 |  |  |
| Model 1 | 1 (reference) | 4.39(1.9-59.90) | 6.50(2.43-17.40) | ＜0.001 |  |
| Model 2 | 1 (reference) | 4.42(1.96-9.99) | 6.88(2.54-18.66) | ＜0.001 | 1.67(1.34-2.07) |
| Incidence rate: per 1000 person-years.  Model 1: adjusted for age, sex;  Model 2: Model 1+ education level, income level, marital status, alcohol consumption and family history of CVD. | | | | | |

| **Table S3.** Distinct effects of health factors and health behaviors on CVD and all-cause mortality (for each exclusion of a controlled factor) | | |
| --- | --- | --- |
|  | **Health factors** | **Health behaviors** |
| **CVD** | 1.75(1.55-1.98) | 1.02(0.87-1.20) |
| **All-cause mortality** | 1.38(1.21-1.58) | 1.18(1.01-1.39) |
| **Stroke** | 1.69(1.47-1.93) | 0.99(0.82-1.18) |
| **Myocardial infarction** | 2.10(1.63-2.69) | 1.11(0.78-1.57) |
| Model: adjusted for age, sex, education level, income level, marital status, alcohol consumption and family history of CVD. | | |

| **Table S4.** Sensitivity analyses of associations between the degree of joint risk factor control and CVD and all-cause mortality | | | | | |
| --- | --- | --- | --- | --- | --- |
|  | **Degree of Risk Factor Control** | | | ***P* value** | **Each Additional Uncontrolled Risk Factor** |
|  | **≥7 Risk Factors** | **5-6 Risk Factors** | **≤4 Risk Factors** |  |  |
| **Participants with CVD or death within the first 2 years of follow-up excluded** | | | | | |
| **CVD** |  |  |  |  |  |
| Model 1 | 1 (reference) | 2.34(1.71-3.20) | 3.08(2.01-4.72) | ＜0.001 |  |
| Model 2 | 1 (reference) | 2.27(1.66-3.11) | 2.99(1.94-4.62) | ＜0.001 | 1.43(1.29-1.60) |
| **All-cause mortality** |  |  |  |  |  |
| Model 1 | 1 (reference) | 1.62(1.20-2.18) | 2.71(1.81-4.07) | ＜0.001 |  |
| Model 2 | 1 (reference) | 1.57(1.17-2.12) | 2.59(1.71-3.92) | ＜0.001 | 1.29(1.15-1.44) |
| **Participants with history of cancer at baseline or onset cancer during follow-up excluded** | | | | | |
| **CVD** |  |  |  |  |  |
| Model 1 | 1 (reference) | 2.29(1.70-3.09) | 3.14(2.09-4.72) | ＜0.001 |  |
| Model 2 | 1 (reference) | 2.24(1.66-3.02) | 3.09(2.04-4.67) | ＜0.001 | 1.44(1.30-1.60) |
| **All-cause mortality** |  |  |  |  |  |
| Model 1 | 1 (reference) | 1.59(1.20-2.12) | 2.82(1.92-4.15) | ＜0.001 |  |
| Model 2 | 1 (reference) | 1.54(1.16-2.06) | 2.68(1.81-3.98) | ＜0.001 | 1.31(1.17-1.46) |
| **A 2-year lag analysis for associations of duration of adherence** | | | | | |
| **CVD** |  |  |  |  |  |
| Model 1 | 1 (reference) | 2.35(1.72-3.21) | 3.07(2.00-4.71) | ＜0.001 |  |
| Model 2 | 1 (reference) | 2.29(1.67-3.13) | 2.99(1.93-4.62) | ＜0.001 | 1.43(1.29-1.59) |
| **All-cause mortality** |  |  |  |  |  |
| Model 1 | 1 (reference) | 1.58(1.19-2.10) | 2.78(1.89-4.08) | ＜0.001 |  |
| Model 2 | 1 (reference) | 1.53(1.15-2.04) | 2.65(1.79-3.93) | ＜0.001 | 1.30(1.17-1.45) |
| Model 1: adjusted for age, sex;  Model 2: Model 1+ education level, income level, marital status, alcohol consumption and family history of CVD. | | | | | |

| **Table S5**. Associations between the degree of joint risk factor control and CVD and all-cause mortality stratified by age or sex | | | | | |
| --- | --- | --- | --- | --- | --- |
|  | **Degree of Risk Factor Control** | | |  |  |
|  | **≥7 Risk Factors** | **5-6 Risk Factors** | **≤4 Risk Factors** | ***P*_interaction_** | **Each Additional Uncontrolled Risk Factor** |
| **CVD** | | | | | |
| **Sex** |  |  |  | 0.67 |  |
| female | 1 (reference) | 2.18(0.82-5.81) | 10.51(1.31-84.40) |  | 2.11(1.27-3.50) |
| male | 1 (reference) | 2.23(1.62-3.05) | 3.02(1.97-4.63) |  | 1.42(1.28-1.58) |
| **age** |  |  |  | 0.34 |  |
| ＜30 | 1 (reference) | 2.24(1.06-4.74) | 3.34(1.02-10.97) |  | 1.48(1.09,2.00) |
| ≥30 | 1 (reference) | 2.22(1.60-3.08) | 3.02(1.93-4.70) |  | 1.43(1.28,1.60) |
| **All-cause mortality** | | | | | |
| **Sex** |  |  |  | 1.00 |  |
| female | 1 (reference) | 1.56(0.72-3.38) | - |  | 1.34(0.80-2.23) |
| male | 1 (reference) | 1.53(1.12-2.08) | 2.66(1.77-4.00) |  | 1.29(1.15-1.44) |
| **age** |  |  |  | 0.88 |  |
| ＜30 | 1 (reference) | 1.10(0.51-2.38) | 1.33(0.29-6.06) |  | 1.12(0.76-1.64) |
| ≥30 | 1 (reference) | 1.60(1.17-2.18) | 2.81(1.85-4.25) |  | 1.31(1.17-1.47) |
| Model: adjusted for age, sex, education level, income level, marital status, alcohol consumption and family history of CVD. | | | | | |

| **Table S6**. Baseline characteristics of the study population (No-missing data and missing data population). | | | |
| --- | --- | --- | --- |
| **Characteristics** | **No-missing data**  **(n=15948)** | **missing data**  **(n=548)** | ***P* value** |
| **Age (y)** | 32.34±5.19 | 32.70±5.15 | 0.12 |
| **Male (%)** | 11923 (74.76) | 355 (64.78) | <0.001 |
| **Education level (%)** |  |  | 0.03 |
| Illiteracy or primary school | 148 (0.93) | 9 (1.71) |  |
| Junior high school | 8280 (51.92) | 248 (47.24) |  |
| Senior high school and above | 7520 (47.15) | 268 (51.05) |  |
| **Income level, ¥/month (%)** |  |  | 0.57 |
| ≤ 800 | 12826 (80.42) | 431 (82.10) |  |
| 800-1000 | 1574 (9.87) | 45 (857) |  |
| ≥ 1000 | 1548 (9.71) | 49 (9.33) |  |
| **Alcohol consumption (%)** |  |  | 0.01 |
| Never | 8167 (51.21) | 302 (57.52) |  |
| Current drinker | 284 (1.78) | 9 (1.71) |  |
| Past | 7497 (47.01) | 214 (40.76) |  |
| **Married individuals (%)** | 14479 (90.79) | 489 (93.14) | 0.07 |
| **Family history of CVD (%)** | 816 (5.12) | 13 (2.37) | 0.003 |
| **Health factors under controlled (%)** |  |  |  |
| Blood glucose | 15540 (97.44) | 271 (49.45) | <0.001 |
| Blood pressure | 12800 (80.26) | 355 (64.78) | <0.001 |
| Blood lipids | 13459 (84.39) | 322 (58.76) | <0.001 |
| Body mass index | 14557 (91.28) | 358 (55.33) | <0.001 |
| **Health behaviours under controlled (%)** |  |  |  |
| Diet health | 4582 (28.73) | 144 (26.28) | 0.21 |
| Nicotine exposure | 10051 (63.02) | 333 (60.77) | 0.28 |
| Physical activity | 14134 (88.63) | 418 (76.28) | <0.001 |
| Sleep health | 15134 (94.90) | 396 (72.26) | <0.001 |
| Values are means (SD) for continuous variables or percentages for categorical variables. | | | |

| **Table S7.** Association between the degree of joint risk factor control and incident CVD and all-cause mortality (including the population with missing data). | | | | | |
| --- | --- | --- | --- | --- | --- |
|  | **Degree of Risk Factors Control** | | | ***P* value** | **Each Additional Uncontrolled Risk Factor** |
|  | **≥ 7 Risk Factors**  (N=7747) | **5-6 Risk Factors**  (N=7570) | **≤ 4 Risk Factors**  (N=1179) |  |  |
| **CVD** | | | | | |
| Events/person-years | 62/114699 | 191/111780 | 43/17119 |  |  |
| Incidence rate | 0.54 | 1.71 | 2.51 |  |  |
| Model 1 | 1 (reference) | 2.26(1.68-3.03) | 2.79(1.87-4.16) | ＜0.001 |  |
| Model 2 | 1 (reference) | 2.22(1.65-2.98) | 2.80(1.87-4.19) | ＜0.001 | 1.37(1.24-1.51) |
| **All-cause mortality** | | | | | |
| Events/person-years | 77/115018 | 164/112678 | 52/1179 |  |  |
| Incidence rate | 0.67 | 1.46 | 2.99 |  |  |
| Model 1 | 1 (reference) | 1.67(1.26-2.21) | 2.87(2.00-4.16) | ＜0.001 |  |
| Model 2 | 1 (reference) | 1.62(1.22-2.15) | 2.75(1.89-4.00) | ＜0.001 | 1.30(1.18-1.44) |
| Incidence rate: per 1000 person-years.  Model 1: adjusted for age, sex;  Model 2: Model 1+ education level, income level, marital status, alcohol consumption and family history of CVD. | | | | | |

| **Table S8.** Association between the degree of joint risk factor control and incident CVD and all-cause mortality (sensitivity analysis of redefining grouping threshold). | | | | | |
| --- | --- | --- | --- | --- | --- |
|  | **Degree of Risk Factors Control** | | | ***P* for trend** | **Each Additional Uncontrolled Risk Factor** |
|  | **≥ 5 Risk Factors**  (N=4445) | **4 Risk Factors**  (N=4345) | **≤ 3 Risk Factors**  (N=7158) |  |  |
| **CVD** | | | | | |
| Events/person-years | 15/65812 | 55/64393 | 215/105463 |  |  |
| Incidence rate | 0.22 | 0.85 | 2.04 |  |  |
| Model 1 | 1 (reference) | 2.59(1.45-4.61) | 4.95(2.89-8.50) | ＜0.001 |  |
| Model 2 | 1 (reference) | 2.50(1.4-4.46) | 5.10(2.96-8.77) | ＜0.001 | 1.64(1.44-1.87) |
| **All-cause mortality** | | | | | |
| Events/person-years | 42/65920 | 62/64654 | 170/406504 |  |  |
| Incidence rate | 0.63 | 0.95 | 1.59 |  |  |
| Model 1 | 1 (reference) | 1.06(0.71-1.59) | 1.42(1.00-5.04) | ＜0.001 |  |
| Model 2 | 1 (reference) | 1.04(0.70-1.55) | 1.41(1.00-2.03) | ＜0.001 | 1.18(1.05-1.33) |
| Incidence rate: per 1000 person-years.  Model 1: adjusted for age, sex;  Model 2: Model 1+ education level, income level, marital status, alcohol consumption and family history of CVD. | | | | | |

| **Table S9.** Association between the degree of joint risk factor control and incident CVD and all-cause mortality: Results from weighted Cox regression analysis. | | | | |
| --- | --- | --- | --- | --- |
|  | **Degree of Risk Factors Control** | | | ***P* for trend** |
|  | **≥ 7 Risk Factors**  (N=7708) | **5-6 Risk Factors**  (N=7239) | **≤ 4 Risk Factors**  (N=1001) |  |
| **CVD** | | | | |
| Model 1 | 1 (reference) | 2.30(1.71-3.10) | 3.20(2.15-4.76) | ＜0.001 |
| Model 2 | 1 (reference) | 2.25(1.67-3.03) | 3.14(2.10-4.70) | ＜0.001 |
| **All-cause mortality** | | | | |
| Model 1 | 1 (reference) | 1.16(1.19-2.10) | 2.79(1.92-4.07) | ＜0.001 |
| Model 2 | 1 (reference) | 1.53(1.15-2.04) | 2.66(1.80-3.90) | ＜0.001 |
| Incidence rate: per 1000 person-years.  Model 1: adjusted for age, sex;  Model 2: Model 1+ education level, income level, marital status, alcohol consumption and family history of CVD. | | | | |
